# Mobile schwannoma of the lumbar spine: A case report and literature review

**DOI:** 10.1097/MD.0000000000034966

**Published:** 2023-08-25

**Authors:** Shaowen Song, Xiaojing Li, Wanqiu Lyu, Liyuan Chen, Binqing Zhang

**Affiliations:** a Medical Imaging Center, Luoyang Orthopedic-Traumatological Hospital of Henan Province (Henan Provincial Orthopedic Hospital), Luoyang, Henan Province, China; b Department of Radiology, The Third Affiliated Hospital of Henan University of Traditional Chinese Medicine, Zhengzhou, Henan Province, China; c Department of Radiology, China-Japan Union Hospital of Jilin University, Changchun, Jilin Province, China.

**Keywords:** case report, lumbar spine, magnetic resonance imaging, mobile schwannoma, surgery

## Abstract

**Background:**

Schwannoma is a benign tumor that originates from the cells of nerve sheaths. The occurrence of mobile schwannoma of the lumbar spine is extremely rare, and when clinicians perform spinal explorations in the expected locations, the results are often negative. This report presents a case of mobile schwannoma in the lumbar spine, aiming to remind doctors to consider the possibility of tumor migration during preoperative planning, thereby avoiding secondary harm to the patient.

**Case report:**

A 48-year-old male patient presented with a 2-year history of lower back pain and numbness in both lower extremities, which has progressively worsened over the past 3 months. An Magnetic resonance imaging scan revealed an oval-shaped lesion located in the L2/3 vertebral segment, which migrated to the L3/4 vertebral segment following contrast enhancement. The patient underwent tumor resection surgery, and the diagnosis of schwannoma was confirmed through pathological examination.

**Conclusion:**

Clinical physicians should increase their understanding of mobile schwannoma of the lumbar spine and utilize imaging techniques to accurately locate the tumor before surgery, in order to avoid unnecessary surgical scope and the risks of additional surgery.

## 1. Introduction

A Schwannoma, also known as a Schwann cell tumor, is a benign tumor originating from Schwann cells of the nerve sheath. It is commonly seen as a single mass with a complete capsule and represents about 25% of all spinal tumors within the spinal canal.^[1]^ Schwannomas are uniformly distributed among various spinal segments and do not show any specific preference for a particular area of the spine. In general, intraspinal Schwannomas rarely metastasize, this article reports a case of a mobile schwannoma of the lumbar spine and provides a systematic review of all cases through PUBMED to improve clinicians understanding of this disease.

## 2. Case report

**Figure 1.**
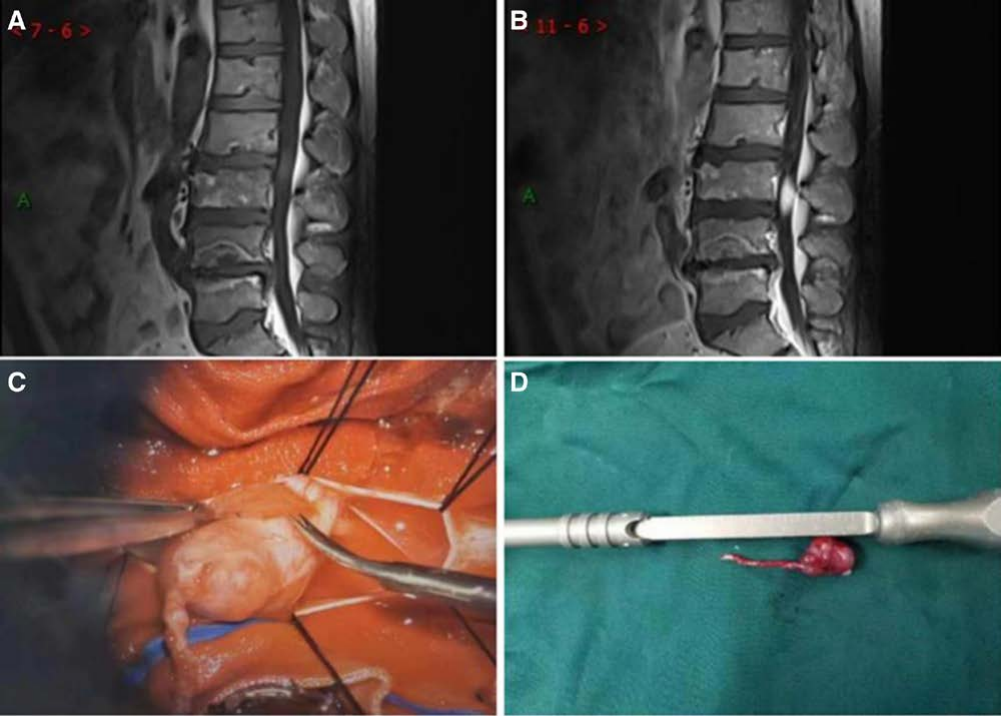
A 48-year-old man with a mobile schwannoma of the lumbar spine. (A) The lesion shows slightly high signal on T1WI and is located at the L2/3 level. (B) Enhancement scan shows obvious enhancement, and the lesion is located at the L3/4 level. (C) Intraoperatively, a spindle-shaped mass is visible within the dura mater, with a bundle of nerves passing through the center. (D) Gross examination after surgery reveals a band-like mass measuring 2cm × 1.5cm × 1cm, with a length of 2.5cm and a diameter of 0.1cm.

A 48-year-old male patient presented to our hospital with a 2-year history of lower back pain and bilateral lower limb numbness, which worsened over the past 3 months. An Magnetic resonance imaging (MRI) of the lumbar spine performed at an outside hospital showed a lesion at the lower border of the L2 vertebral body within the spinal canal. Physical examination revealed slightly hyperactive biceps and triceps tendon reflexes and bilateral Hoffmann signs (+). The lumbar lordosis was flattened, and there was tenderness and right-sided radicular pain upon palpation of the L5/S1 spinous process and paraspinal area, radiating to the right buttock and posterior thigh. Bilateral knee tendon reflexes were reduced, and the dorsiflexion of the foot and ankle muscles was weakened to approximately grade IV (Medical Research Council grading system). Electrophysiological testing showed evidence of left lower limb radiculopathy involving the L5 root and right anterior tibial muscle. Laboratory tests showed no abnormalities. MRI plain scans showed an elliptical abnormal signal in the spinal canal at the L2/3 level, with slightly high signal on T_1_WI and slightly high signal on T_2_WI and fat-suppression sequence (Fig. 1A). Contrast-enhanced scans showed that the lesion was located in the spinal canal at the L3/4 level and was significantly enhanced (Fig. 1B).

**Figure 2.**
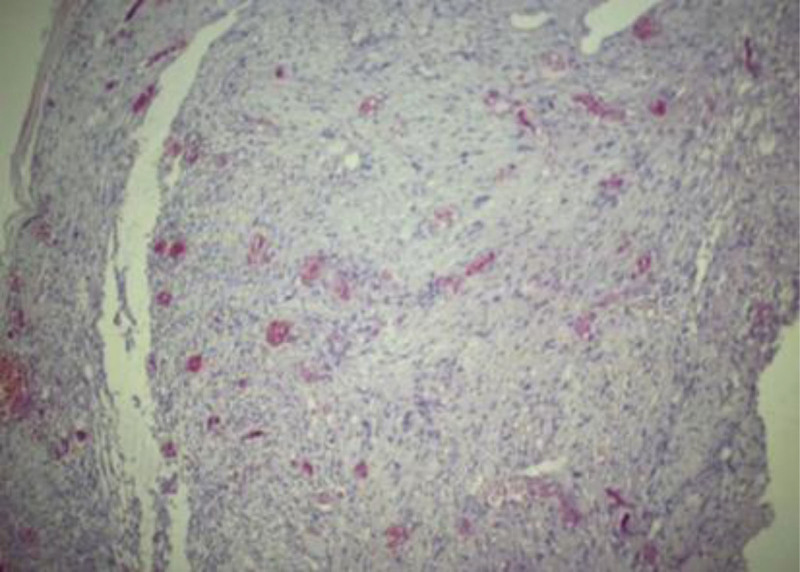
Histopathological examination shows spindle or short-spindle-shaped tumor cells, some of which are arranged in a whirlpool pattern (HE, ×100).

Through a posterior midline approach, a tumor resection was performed, along with laminectomy and durotomy at the L3/4 level. The cauda equina was explored, and a spindle-shaped mass was identified with a bundle of nerves passing through the center (Fig. 1C and D). The affected nerves were cauterized using bipolar electrocoagulation, and the mass was dissected with micro scissors and sent for pathological examination. Pathology revealed a gray-brown, firm mass with fibrous bands on the cut surface. Under the microscope, the tumor cells were spindle or short-spindle-shaped, with some arranged in a swirling pattern (Fig. 2). Immunohistochemical analysis showed S-100 (+), SOX10 (+), EMA (−), Ki-67 (approximately 1% +), and Vimentin (+). The pathological diagnosis was (lumbar spinal canal) schwannoma. Luoyang Orthopedic-Traumatological Hospital of Henan Province (Henan Provincial Orthopedic Hospital) exempted this study from review, and informed consent from the patient has been obtained.

## 3. Discussion

In 1963, Wortzman reported the first case of an intradural-mobile tumor, which was a myxopapillary ependymoma with a distended filum terminale.^[2]^ In 1974, Tomimatsu reported the first case of an intradural-mobile schwannoma, and since then, 29 cases have been reported (Table 1).^[3–25]^ In this case, there were 25 males and 5 females with an average age of (49.62 ± 13.88) years, and 1 case with no reported age and gender. The most common location of the lesion was in the lumbar region (60%), followed by the thoracic region (17%), the thoracolumbar junction (13%), the cervicothoracic junction (7%), and the cervical region (3%). It was observed that all tumors migrated rostral (43%), caudal (43%), or moved back and forth within the spinal canal (13%). The migration distance was within 1 vertebral body in 63% of cases, between 1 and 2 vertebral bodies in 27%, and bigger than 2 vertebral bodies in 10%. One case resulted in entrapment due to migration toward the caudal region after trauma. Clinical symptoms mainly included lower back pain and radiating leg pain with numbness. The activity of the tumor was rarely evident in clinical presentation, and only 3 cases have been reported in the literature, in which symptoms worsened after tumor migration and were related to changes in position or posture, and Valsalva maneuver.^[4,6,9]^

**Table 1:** Summary of previously published cases of intradural-mobile schwannoma.

Author year	Case	Age gender	Initial location	Move location	Distance (vertebral body)	Migration direction
Tomimatsu et al, 1974^[3]^	1	47 M	C4-C6	C2-C4	2	Rostral
Hollin et al, 1978^[4]^	1	56 W	L1	L4/L5	3.5	Caudal
Pau, et al, 1982^[5]^	1	50 W	L2	L3	1	Repeated movement
Tavy et al, 1987^[6]^	1	45 M	L2	L3/4	1.5	Caudal
Isu et al, 1989^[7]^	3	51 W	T11/T12	T12/L1	1	Caudal
		42 M	T12/L1	L1/L2	1	Caudal
		52 M	L1/L2	L2	0.5	Caudal
Satoh et al, 1991^[8]^	1	66 M	L1	T12	1	Rostral
Namura et al, 1993^[9]^	1	51 M	T1/T5	T6/T10	5	Caudal
Varughese et al, 1997^[10]^	2	65 M	L5	L3	2	Rostral
		78 M	L2/3	L1	1.5	Repeated movement
Iizuka et al, 1998^[11]^	1	48 M	C7/T1	T1/T2	1	Caudal
Friedman et al, 2000^[12]^	1	28 M	L4	L3	1	Rostral
Marin-Sanabria et al, 2007^[13]^	2	27 M	L1	L2	1	Caudal
		41 M	L2/L3	L1/L2	1	Rostral
Kim et al, 2010^[14]^	3	45 M	L3/L4	L2/L3	1	Rostral
		32 M	T10/T11	T11	0.5	Caudal
		27 M	L3/L4	L2	1.5	Rostral
Stuart et al, 2010^[15]^	1		L4	L3	1	Rostral
Sasaki et al, 2011^[16]^	1	56 M	L4/L5	L3/L4	1	Rostral
Khan et al, 2012^[17]^	1	52 M	T11/T12	T6/T7	5	Repeated movement
Terada et al, 2016^[18]^	1	68 M	C5/C7	C6/T1	1	Repeated movement
Toscano et al, 2016^[19]^	1	40 M	T11/T12	T12/L1	1	Caudal
Kothari et al, 2017^[20]^	1	70 M	L2/L3	L3/L4	1	Caudal
Onishi et al, 2018^[21]^	1	49 M	L3	L4/L5	1.5	Caudal
Shunjie Jia et al, 2019^[22]^	1	64 W	T5/T6	T3/T4	2	Rostral
Hamabe et al, 2019^[23]^	1	48 W	L4/S1	L2/L4	2	Rostral
Sarikaya et al, 2020^[24]^	1	68 M	L3	L2/L3	0.5	Rostral
Honda et al, 2020^[25]^	1	25 M	T10/T11	T10/T11	10 mm	Rostral
Current case	1	48 M	L2/L3	L3/L4	1	Caudal

Several hypotheses have been proposed to explain the reasons for intradural tumor migration, and literature reports have observed redundancy of the cauda equina during myelography.^[5,10,14,23,24]^ In most cases, there are no attachments within the dura mater, and the only restriction for the tumor is the roots connecting the 2 ends.^[14]^ Since there is no spinal cord in the lumbar region, the nerve roots can float in the cerebrospinal fluid, and tumors attached to the redundant cauda equina can have greater mobility. Moreover, when the lumbar spine and buttocks are in an extended position or the weight of the tumor is significant, it can cause the nerve roots to become lax, which can increase the mobility of the attached tumor.^[11,26]^ Change in posture is also another reason, Isu et al^[7]^ observed tumor activity after gradually changing patient posture and performing repeated MR imaging. Pau et al^[5]^ also observed migrating tumors after changing patient posture following myelography. Therefore, having the patient perform provocative movements, such as back extension, flexion, and jumping, may help detect tumor mobility. Spinal cord deformities caused by extradural tumors may cause corresponding abnormal dilation of the subarachnoid space, 3 cases have been reported in the past, all occurring in the cervical-thoracic region.^[9,11,18]^ Comparable to the lumbar region, the cervical-thoracic region has a narrower subarachnoid space and shorter nerve roots, so the degree of dilation is often smaller, which may also explain why mobility tumors in the cervical-thoracic region are rarer than in the lumbar region. Some authors have proposed that the Valsalva maneuver can cause tumor movement, and our group has reported 6 cases, all of which moved caudal.^[7,9,13,14]^ Valsalva maneuver is an action that increases intra-abdominal and intrathoracic pressure by muscle contraction and breath-holding and can be triggered during forceful urination or defecation.^[27]^ The reason why all 6 patients had tumors moving downward may be due to an increase in intra-abdominal pressure causing the tumor to move downward. It has also been reported that the push force of the contrast agent during myelography can cause tumor migration.^[5,6]^ However, myelography has been replaced by MRI in current preoperative evaluations, and myelography was not used in this case.

Accurate localization of a tumor before entering the subdural space is crucial for the removal of a mobile intradural tumor, which requires a durotomy and laminectomy. Hollin and Terada reported performing additional laminectomies, with Hollin resection reaching the T12 to L5 level,^[4,18]^ and several authors have reported experiences with second surgeries.^[6,10,14,17,22]^ Accurate preoperative localization can reduce the extent of laminectomy and durotomy, as well as the risk of infection and complications for the patient. Currently, various intraoperative or perioperative examinations play a significant role in confirming the precise location of the tumor, such as intraoperative ultrasound, intraoperative MRI, and myelography.^[6,12,13]^ In the past, myelography was previously considered the gold standard for diagnosis, but the injection of contrast agents can only be completed before opening the dura, and the injection force itself can cause tumor movement, leading to its reduced use. Intraoperative MRI can provide excellent resolution and real-time guidance, but most hospitals do not have this equipment. Friedman confirmed that intraoperative ultrasound helps locate extradural tumors before opening the dura, but it requires higher skills from the operator.^[12]^ Kim suggested that the Valsalva maneuver may help reveal the tumor in situations where there is a lack of imaging modality during surgery.^[14]^

This study has the following limitations; This study did not conduct long-term follow-up of patients, which limited the evaluation of long-term efficacy and side effects; Patient individual differences in case reports may be large, including age, gender, health status, etc, which may affect the interpretation and promotion of results; Generally speaking, positive or abnormal cases are easier to report and publish, while ordinary or negative cases may be ignored, resulting in publication bias.

## 4. Conclusion

We reported a case of mobile schwannoma of the lumbar spine, which is a rare disease that clinicians should be aware of. Accurate preoperative diagnosis is particularly important to avoid unnecessary laminectomy and repeated surgeries. Intraoperative MRI, intraoperative ultrasound, myelography, and Valsalva maneuver can all help detect the lesion. When the surgeon does not find the tumor at the site of the dural opening, the possibility of lesion migration should be considered.

## Author contributions

**Data curation:** Wanqiu Lyu.

**Investigation:** Xiaojing Li.

**Supervision:** Liyuan Chen.

**Writing – original draft:** Shaowen Song.

**Writing – review & editing:** Binqing Zhang.
